# Measurement Indicators of Age-Friendly Communities: Findings From the AARP Age-Friendly Community Survey

**DOI:** 10.1093/geroni/igaa057.166

**Published:** 2020-12-16

**Authors:** Kyeongmo Kim, Tommy Buckley, Denise Burnette, Sunghwan Cho

**Affiliations:** 1 Virginia Commonwealth University, Richmond, Virginia, United States; 2 VCU, Richmond, Virginia, United States

## Abstract

Cities and counties worldwide have adopted the concept of “age-friendly communities” to promote the well-being of older adults. An age-friendly community is a place that provides a safe and affordable built environment and a social environment that encourages older adults’ participation. A major limitation in this field is the lack of valid and reliable measures of age-friendly communities. This study used data from the AARP 2016 Age-Friendly Community Surveys (N=3,652 adults ages 65 and older). This study included 57 indicators of age-friendliness (e.g., housing, transportation, public space, civic engagement, volunteering, community, and health services); socio-demographic characteristics; and health-related characteristics. We randomly split the sample into two subsamples for confirmatory factor analysis (CFA) (n=1,682) and structural equation modeling (SEM) (n=1,682). The CFA resulted in a three-factor structure to measure age-friendly communities: built environment, transportation, and social environment. Model fit indices were acceptable (χ²(44)=14204.09; p<.001; RMSEA=.067; CFI=.912; TLI=.909; SRMR=.05). Internal reliability of the three-factor structure was excellent ranging from .93 to .96. The SEM model showed that older adults living in a community with a greater built environment (β=.119; p=.001) and the social environment (β=.199; p<.001) had higher levels of physical health, after adjusting for all other variables. The findings highlight that the measures of age-friendly communities are reliable and valid. Practitioners and policymakers should work on improving both the built and the social environment to promote the well-being of older adults. The findings also suggested that researchers can use the measures as an evaluation tool for an age-friendly community initiative.

